# Glasses as sources of condensed phosphates on the early earth

**DOI:** 10.1186/1467-4866-15-8

**Published:** 2014-06-06

**Authors:** Nils G Holm

**Affiliations:** 1 Department of Geological Sciences, Stockholm University, Stockholm, Sweden

**Keywords:** Condensed phosphates, Pyrophosphate, Trimetaphosphate, Early Earth, Archean, Hadean, Ultramafic glass, Basaltic glass, Komatiite, Oceanic basement

## Abstract

Procedures for the analysis of phosphorus in geological material normally aims for the determination of the total amount of P expressed as orthophosphate $\left({\text{PO}}_{4}^{3-}\right)$ or the differentiation between inorganic and organic P. This is probably due to analytical difficulties but also to the prevalent opinion that the chemistry of phosphorus in geological environments is almost entirely restricted to the mineral apatite. Because of the low solubility of apatite it is, therefore, commonly argued that little P was around for prebiotic chemistry and that pre-biological processes would essentially have had to do without this indispensable element unless it was provided by alternative sources or mechanisms (such as reduction and activation by lightning or delivery to Earth by celestial bodies). It is a paradox that the potential existence of reactive phosphorus compounds, such as the mineral schreibersite - iron phosphide, in geological material on Earth is seldom considered although we are aware of the existence of such compounds in meteorite material. The content of Al_2_O_3_ in rocks appears to be important for the speciation of phosphorus and for how strongly it binds to silicates. In general, low alumina seems to promote the existence of isolated charge-balanced phosphorus complexes.

## Introduction

It is conventional wisdom that phosphorus has always been a scarce element on Earth, that the little P that was around was locked up in insoluble apatite, and that no P in the form of condensed phosphates, like pyrophosphate, occurs in the geosphere [1-3]. This is not necessarily true. We know little of the speciation of P in the lithosphere, especially in melts and non-crystalline materials like glasses and gels [4]. The content of Al_2_O_3_ appears to be important for phosphorus chemistry, since increases in the alumina/alkali ratio leads to a decreased effect of phosphorus on silicate polymerization [5]. The suggestion that condensed phosphates are dissolved in glasses is supported by experiments carried out by Cody, Mysen, and colleagues [5-7]. Their studies showed that pyrophosphate and trimetaphosphate ((P_3_O_9_)^3-^) are stable in alkaline glasses at low concentrations of Al_2_O_3_. At about 2 mol% Al_2_O_3_ the phosphate speciation is dominated by pyrophosphate. At 5 mol% Al_2_O_3_ trimetaphosphate appears to be present. Phosphorus shows a stronger preference for the basaltic – mafic – member of coexisting immicible granitic and basaltic melts [8]. Yamagata and coworkers [9] have, accordingly, reported the occurrence of pyrophosphate (0.45 μM) and triphosphate (0.37 μM) in fumaroles presumably formed through partial hydrolysis of P_4_O_10_ emanating from basaltic melts of Mount Usu on the island of Hokkaido, Japan. Ultramafic lavas with an even lower content of SiO_2_ (about 40–45 wt%) than basalt (45-52 wt%), a CaO:Al_2_O_3_ ratio >1, and more than 18 wt% MgO, has been given the term komatiites [10,11]. Abundant deposition of ultramafic volcanic glasses due to komatiitic volcanism in the Hadean and early Archean is probable [12,13], and oceanic crust is likely to have been komatiitic and fed from komatiitic liquids [14]. Ultramafic glasses may have played an important role in prebiotic chemistry of P on the early Earth.

## Condensed phosphates in prebiotic chemistry

Keefe and Miller discussed about 20 years ago whether condensed phosphates like pentavalent pyrophosphate ((P_2_O_7_)^4-^) were ever likely prebiotic reagents on Earth [2]. At the time they argued that a widespread occurrence of protonated orthophosphates, which are known to serve as precursors of pyrophosphate, is doubtful in nature (cf. [15]), and that heating of such protonated phosphates in a closed system does not give polyphosphates because water cannot escape. However, pyrophosphate could have been formed during early subduction of oceanic lithosphere by dehydration of known protonated orthophosphates like whitlockite (Ca_18_Mg_2_H_2_(PO_4_)_14_) and newberyite (MgHPO_4_^.^3H_2_O) [16-18]. Whitlockite forms in a sterile (non-biotic) seawater medium in the pH range 7–9 [19]. Newberyite crystallizes at lower pH [20]. Prior to the development of life on Earth, newberyite may have preceded organogenic apatite as the dominant inorganic phosphate mineral being formed in the primordial ocean floor [16,21]. The key to the pyrophosphate formation in these geological environments is low water to rock ratio or low rate of hydrolysis, i.e. low local activity of water, which has the effect that the water can be removed [4]. Pyrophosphate may have preceded ATP (adenosine triphosphate) as energy ‘currency’ in connection with the origin and early evolution of life on Earth [22,23]. It is possible that pyrophosphate was originally incorporated into prebiotic systems because of its energy-transfer capacity and that the information coding function of P as a constituent of polynucleotides is a secondary property [24]. Another condensed phosphate, the ring-formed trimetaphosphate ((P_3_O_9_)^3-^), has, on the other hand, been shown to be the most effective condensing agent among polyphosphates, particularly because it can serve as a phosphorylating agent reagent in strongly alkaline aqueous solutions (pH 12) [18,25-28]. Such high pH values can be found in, for instance, the fairly recently discovered modern sediment-starved subduction zones with serpentinite mud volcanoes, like the Mariana Trench [29,30]. Serpentine mud volcanoes similar to those of the Mariana forearc next to the trench occur at Isua, Greenland, and have been dated to early Archean (3.81-3.70 Ga) [31]. Prieur has shown that it is possible to phosphorylate the carbohydrate ribose with trimetaphosphate to ribose-5-phosphate – which is a central part of nucleotides like ATP and RNA (ribonucleic acid) - in the presence of borate [32,33]. According to Prieur, pyrophosphate does not appear to have as good phosphorylating effect.

## Delivery of phosphorus by meteorites

Since Keefe and Miller published their paper in 1995, Pasek and Block [34] have analyzed lightning-derived glass compounds, termed fulgerites, and shown that phosphate can be reduced to phosphite and phosphide by cloud-to-ground lightning. The reduction of phosphate to phosphite by lightning discharge had been shown a few years earlier in laboratory experiments carried out by Glindemann et al. [35]. Pasek and co-workers also suggested that phosphite derived from the iron-nickel phosphide mineral schreibersite ((Fe,Ni)_3_P) in meteorites was a plausible reagent in the prebiotic synthesis of phosphorylated biomolecules on the early Earth [36-38]. Schreibersite is particularly abundant in some iron meteorites [39]. The phosphite that Pasek and co-workers postulate to have been prebiotically active would be an oxidation product of phosphide that was delivered to Earth during the Hadean-Archean heavy meteorite bombardment >3.9 Ga [37]. This scenario is a potential solution of the ‘phosphate problem’ as discussed by Schwartz [40,41], i.e. solubilization of phosphate compounds is necessary before activation can occur. Schreibersite oxidizes slowly in contact with fluid water as the surrounding mineral matrix gets weathered, and forms several phosphorus species of mixed oxidation states like orthophosphate, pyrophosphate, hypophosphate, phosphite, etc. [36-38,42,43].

## Condensed phosphorus compounds in minerals and glasses on earth

Environments with condensed phosphorus compounds are not absent on our own planet, although such compounds are normally not searched for. Standard analytical procedures of P in geological material include the hydrolysis of all potential forms of P to orthophosphate $\left({\text{PO}}_{4}^{3-}\right)$. The first occurrence on Earth of a natural pyrophosphate mineral, canaphite, was not reported in the scientific literature until 1985 [44,45]. The second mineral, wooldridgeite, was discovered in 1999 [46]. The canaphite was found on crystals of the zeolite mineral stilbite in cavities of Triassic basalts in New Jersey, USA, whereas wooldridgeite was found in an unconformity between Precambrian igneous rocks and overlying Cambrian sedimentary rocks in Warwickshire, England. The reason why such minerals were not found earlier maybe reflects discussions in the chemistry community. It was actually not generally accepted in chemistry that polyphosphates larger than pyrophosphate existed until mid-20th century [47]. The idea of triphosphate as a crystalline entity was, for instance, not accepted until around 1940 [47].

In modern oceanic sediments in areas of low-temperature hydrothermal activity phosphate is strongly enriched as authigenic phases in the basal sedimentary layer on top of the basaltic basement, with the source of phosphorus being primarily the basalts underneath [48]. The phosphorus reported in the study of Bodeï and co-workers was determined in bulk and occurred in association with zeolites and Fe-Mn oxide hydroxides. Silicate minerals can account for as much as 25–30% of the total content of P in igneous and metamorphic rocks [49], i.e. the rocks may be undersatured with respect to separate phosphate minerals. Studies have shown that partitioning of phosphorus between silicate solids preferentially favors glasses, alkaline glasses in particular, relative to olivine, amphibole, and clinopyroxene [50]. Experiments by Milman-Barris and co-workers show that glass adjacent to olivine phenocrysts is enriched relative to the far-field glass by up to 20% in P_2_O_5_, so the glass appears to extract phosphorus from the olivine [51]. Glass of phosphate is widely distributed in the lithospheric mantle and is formed during rapid quenching of mantle material [52].

Phosphorus of different speciation may be released from the basalts and precipitated as secondary protonated phases during water convection and weathering in the surface parts of marine basement. Magnesium pyrophosphate can be formed easily under mild hydrothermal conditions (165-180°C), like those existing in subduction zones, from protonated Mg salts and orthophosphate, for example newberyite [53-55]. The reason is probably that the size of the Mg^2+^ ion makes it possible to simultaneously coordinate and stabilize negatively charged oxygen of two adjacent phosphorus atoms, i.e. Mg(II) has the function of being a ‘bidentate clamp’ [56]. Condensed phosphates have stronger binding energies to hydroxide minerals (e.g. brucite, smectite) than orthophosphate [17]. It has also been shown that the presence of free Fe(II) ions as well as Fe(II) minerals retard the hydrolysis of pyrophosphate [57]. Therefore, it is likely that any condensed phosphates existing in oceanic basement will stay attached to authigenic minerals until the plate is processed in a subduction zone [23]. Nitschke and Russell have proposed that pyrophosphate is dissolved in basaltic glasses and is released upon alteration of the glass into palagonite [58], i.e. a metabasite which contains a mixture of palagonitized glass, authigenic minerals like smectite, corrensite, zeolites, carbonates, and Fe-Ti oxides and phosphates, as well as primary minerals like plagioclase feldspars, clinopyroxene and olivine [59]. The rate of dissolution of phosphate glass is reduced with increased proportions of multiply charged metal ions like Mg(II) [47]. It has, on the other hand, been known for quite some time that the presence of divalent cations like Mg(II) accelerates the formation of ring-formed metaphosphates from polyphosphates [60]. This is probably due to greater ease of chelation within the polyphosphate chains than at the chain ends.

Mitsis and Economou-Eliopoulos concluded in their study of hydroxylapatite associated with massive magnetite in the Othrys ophiolite complex, Greece, that the apatite was formed from re-mobilization of phosphorus and deposition during circulation of hydrothermal fluids [61]. Even though ridge-axis and ridge-flank hydrothermal processes in the ocean floor are estimated to remove about 50% of the global input of P into oceanic crust [62], hydrothermal fluids are obviously able to re-distribute P within hydrothermal systems. However, it is not yet known whether the ‘primary source’ of P after removal from seawater exists entirely in the form of orthophosphate, is bound to the minerals of the mafic rocks, or occur as more soluble condensed P species.

## Conclusions

Phosphorus is an extremely important element for the biological coding of information as well as for the transfer of energy and information in all living organisms on Earth. It is possible that the energy-transfer capacity of phosphorus compounds is a primary property for life processes and that the information coding function is secondary. It is often claimed that phosphorus is a rare and inaccessible element on our planet and that the earliest biosphere, therefore, would have been almost devoid of this indispensable element unless it was provided by alternative sources or mechanisms, such as activation by lightning or delivery as more accessible reduced phosphorus species in iron meteorites. Marine scientists often emphasize that we know more of the surface of the other terrestrial planets than we know of the ocean floor on Earth. This may be true also for the grand scale element cycling of essential nutrients in oceanic basement. The building blocks of the first biochemicals like phosphorus may, after all, have been around and available for prebiotic chemistry during Earth’s entire life span.

## Competing interests

The author declares no competing interest.
